# Opioids With or Without Low-Dose Naloxone During the Perioperative Period: A Systematic Review With Meta-Analysis

**DOI:** 10.1155/prm/8380502

**Published:** 2025-02-23

**Authors:** Benyu Mao, Xian Wang, Xianping Zhang, Min Chen

**Affiliations:** ^1^Department of Colorectal Surgery, The First Affiliated Hospital of Yangtze University, Hangkong Street 8, Jingzhou, Hubei, China; ^2^Department of Pharmacy, The First Affiliated Hospital of Yangtze University, Hangkong Street 8, Jingzhou, Hubei, China

**Keywords:** efficacy, meta-analysis, naloxone, perioperative period, safety

## Abstract

**Objectives:** The aim of this systematic review and meta-analysis from randomized controlled trials is to assess opioids with or without low-dose naloxone during the perioperative period at pain intensity and opioids-related adverse events.

**Methods:** We searched of Medline, Embase, International Clinical Trials Registry Platform, and the Cochrane Library up to May 31, 2023. We included randomized controlled trials (RCTs) of low-dose naloxone combined with opioids in adults reporting pain intensity or opioid-related adverse event during the perioperative period.

**Results:** A total of 18 RCTs with 1784 participants were included. We could not reach a consistent conclusion for pain intensity due to high heterogeneity. High certainty evidence showed that low-dose naloxone combined with opioids reduced the risk of nausea (relative risk (RR): 0.82 and 95% confidence interval (CI): 0.70–0.96), cough (RR: 0.52 and 95% CI: 0.30–0.90) and postoperative nausea and vomiting (RR: 0.58 and 95% CI: 0.40–0.80). Moderate certainty evidence showed that low-dose naloxone combined with opioids did not reduce vomiting, urinary retention, sedation, dizziness, respiratory depression, headache, drowsiness, shivering, skin itch, hypotension, and sweating.

**Conclusions:** Our findings show that the use of low-dose naloxone in combination with opioids can lower the risk of somnolence and coughing, postoperative nausea, and vomiting.

## 1. Introduction

Effective pain management can help to alleviate patients' tension and anxiety, promote early oral intake, and recover bodily functions [1]. Recommendations released in 2023 by the National Comprehensive Cancer Network included using opioids to improve overall function and quality of life [2]. Opioids are highly effective treatment for moderate to severe pain and continue to be essential in improving perioperative pain, even when multimodal analgesia or other analgesic medications are utilized [3]. Opioid therapy has been found to have several adverse effects, such as constipation, vomiting, pruritus and nausea, which are reported to be significant. Therefore, minimizing opioid-related adverse effects while maintaining its analgesic effect is a crucial health concern. Naloxone is an opioid receptor antagonist and has been used to treat respiratory depression associated with opioid overdose. Several studies suggest that administering low-dose naloxone can mitigate the risk of adverse effects associated with opioids, while preserving their analgesic properties [4, 5] whereas some studies hold different opinions [6, 7]. Previously published systematic reviews and meta-analyses have summarized the naloxone treatment on opioid-related side effects [8, 9]. Results from a recent meta-analyses in 2017 report reductions in postoperative nausea and vomiting with low-dose naloxone [8]. However, many trials reported pain intensity or opioids-induced adverse events with naloxone during the perioperative period since 2017. To our knowledge, no large trial or meta-analysis has assessed the potential benefits. The efficacy and safety of using combination opioids with low-dose naloxone during the perioperative period should be updated. The current systematic review and meta-analysis from randomized controlled trials aims to evaluate opioids with or without low-dose naloxone during the perioperative period at pain intensity and opioids-related adverse events.

## 2. Materials and Methods

We reported this systematic review and meta-analysis according to preferred reporting items for systematic reviews and meta-analysis (PRISMA 2020) [10]. The study protocol was prospectively registered in PROSPERO (CRD42023391769).

### 2.1. Data Sources and Searches

We conducted a systematic search of Medline, Embase, International Clinical Trials Registry Platform, and the Cochrane Central Register of Controlled Trials databases for randomized controlled trials involving naloxone up to May 31, 2023. We used the following search terms: “naloxone” AND (“opioid∗” OR “opioid receptor agonist” OR “morphine” OR “fentanyl” OR “remifentanil” OR “sufentanil” OR “hydromorphone” OR “methadone” OR “oxycodone” OR “hydrocodone” OR “oxymorphone” OR “codeine” OR “tramadol” OR “tapentadol” OR “dezocine” OR “pentazocine” OR “butorphanol”). MeSH and free terms were both used to identify eligible trials. No language restriction was applied. To ensure all eligible studies were included, reference lists of relevant articles were manually screened in addition to the initial online search. Previously published meta-analyses were also used as references of the studies sources [8, 9].

### 2.2. Eligibility Criteria

For this subject paper, trials were included if they met the following criteria: (1) research type: randomized controlled trials; (2) research subjects: trials were randomized design with subjects > 18 years; and (3) interventions: patients received either an opioid receptor agonist with low-dose naloxone or just an opioid receptor agonist during the perioperative period. Exclusion criteria included (1) duplicate publications, (2) appropriate data could not be extracted or calculated, and (3) patients received an opioid receptor agonist or naloxone not by vein input.

### 2.3. Data Extraction and Outcome Assessments

Two reviewers independently identified studies and extracted relevant data. We solved discrepancies by consensus. We abstracted data on characteristics of trials and participants (age, ample sizes, operation name, name of opioid or naloxone with doses, and outcomes). The outcomes were the composite of pain intensity (visual analog scale (VAS) pain scores following surgery) and opioid-related adverse events. Outcomes were analyzed according to the intention-to-treat principle.

### 2.4. Risk of Bias and Certainty of Evidence

Two investigators independently used Cochrane tool for appraising the risk of bias in randomized trials (RoB 2.0) [11]. Risk of bias assessment was done across the randomization process, intended interventions, missing outcome data, measurement of the outcome, and selection of the reported result. Each of the RoB domains was as “low risk of bias,” “some concern,” or “high risk of bias.” We solved discrepancies by consensus and used Grading of Recommendations, Assessment, Development, and Evaluations (GRADEs) to assess the certainty of evidence [12].

### 2.5. Data Synthesis and Analysis

We used the relative risk (RR) with 95% confidence intervals (CIs) to describe the effect size of dichotomous outcomes and the mean between group differences with 95% CIs for continuous outcomes. We examined heterogeneity among studies with the Cochrane *Q* test and the *I*^2^ statistic (0%–25% low heterogeneity, 25%–50% moderate heterogeneity, 50%–75% substantial heterogeneity, and 75%–100% high heterogeneity). We performed meta-analysis using Stata 14. We used the random effect model to calculate the pooled means, RR, and 95% CI. All statistical tests were two-sided, and statistical significance was defined as a *p* value of less than 0.05.

## 3. Results

### 3.1. Study Selection and Characteristics of the Studies Included

**Figure 1:**
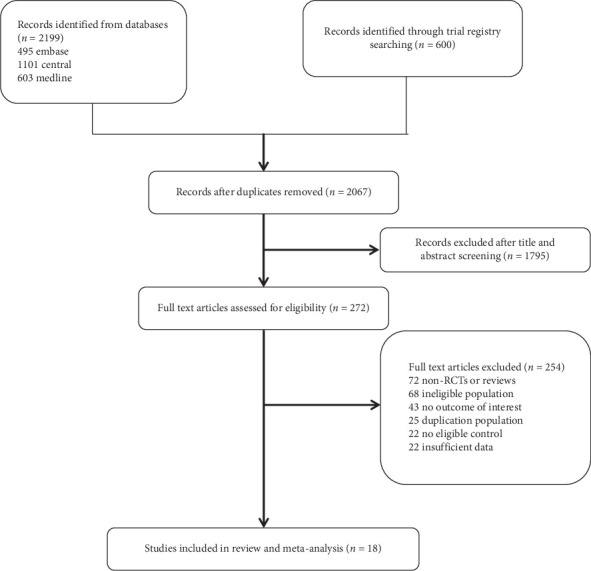
PRISMA flow diagram.

The search and selection process is illustrated in Figure 1. We identified 2799 records through a comprehensive electronic search, 732 of which were duplicates and removed. After screening based on titles and abstracts, the full text of 272 articles was subsequently reviewed. Eighteen trials (involving 1784 participants) were included in the present meta-analysis [4–6, 13–27].  Table 1 shows characteristics of the clinical trials. Of the 18 trials in present meta-analysis, seven trials used morphine, three trials used remifentanil, three trials used fentanyl, three trials used sufentanil, one trial used tramadol, and one trial used butorphanol.

### 3.2. Risk of Bias

**Figure 2:**
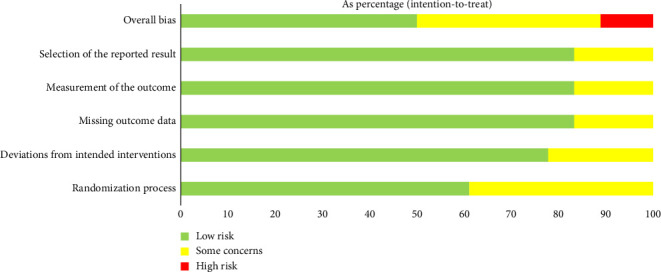
Risk of bias using the Cochrane risk tool.

The risk of bias of eligible trials was shown in Figure 2. Of the 18 completed trials, nine (50%) had low risks or some concerns of bias across all five domains evaluated. A low risk of randomization process was found in 11 studies, a low risk of deviations from intended interventions in 13 studies, a low risk of missing outcome data in 15 studies, a low risk of measurement of outcome in 15 studies, and a low risk of selection of reported results in 15 studies.

### 3.3. Pain Intensity

Eight trials used VAS to rate the intensity of the pain [5, 15, 17, 19, 21, 23, 24, 26]. Due to high heterogeneity (*I*^2^ = 91.8%) among the included trials for pain intensity, we conducted a systematic review without meta-analysis for pain intensity. Five of these trials reported that naloxone significantly lowered pain severity when measured by VAS [5, 17, 19, 21, 23]. However, the administration of naloxone had no overall effect on postoperative VAS pain scores compared with control when assessed by three studies [15, 24, 26].

### 3.4. Adverse Events

Seventeen trials enrolling 1704 participants determined the opioids-related adverse events of low-dose naloxone combined with opioids during the perioperative period. The type of adverse events varied noticeably among trials. Vomiting and nausea were reported as the two most prevalent adverse events in 10 trials.

#### 3.4.1. Nausea

Ten trials determined the opioids-related adverse events of naloxone combined with opioids for nausea. In comparison with control, low-dose naloxone combined with opioids were associated with an decrease in the risk of an adverse event (RR: 0.82, 95% CI: 0.70–0.96, *I*^2^ = 0%, and *p*=0.01; 1072 participants; high certainty evidence).

#### 3.4.2. Vomiting

Ten trials determined the opioids-related adverse events of naloxone combined with opioids for vomiting. In comparison with control, low-dose naloxone combined with opioids did not increase the risk of experiencing an adverse event (RR: 0.86, 95% CI: 0.70–1.06, *I*^2^ = 0%, and *p*=0.16; 1089 participants; moderate certainty evidence).

#### 3.4.3. Postoperative Nausea and Vomiting (PONV)

Five trials reported PONV as a single outcome. When compared with control, low-dose naloxone combined with opioids were associated with an decrease in the risk of an adverse event (RR: 0.58, 95% CI: 0.40–0.80, *I*^2^ = 0.5%, and *p* < 0.05; 434 participants; high certainty evidence).

#### 3.4.4. Pruritus

Moderate certainty evidence showed that low-dose naloxone combined with opioids do not decrease the risk of any adverse event (RR: 0.75, 95% CI: 0.55–1.02, *I*^2^ = 0%, and *p*=0.07; 7 trials, 822 participants).

#### 3.4.5. Others

Compared with control, no differences were found with low-dose naloxone combined with opioids in the risk of experiencing urinary retention, sedation, dizziness, respiratory depression, headache, drowsiness, shivering, skin itch, hypotension, and sweating (Table 2). However, low-dose naloxone combined with opioids was associated with decrease in the risk of somnolence and cough (Table 2).

## 4. Discussion

In this systematic review and meta-analysis, we investigated low-dose naloxone combined with opioids in adults at pain intensity or opioid-related adverse event during the perioperative period. This meta-analysis provides moderate and high certainty evidence that low-dose naloxone combined with opioids, when added to perioperation, reduced nausea, PONV, somnolence, and cough. Low to high certainty evidence showed that low-dose naloxone combined with opioids might not decrease the risk of adverse events, such as vomiting, urinary retention, sedation, dizziness, respiratory depression, headache, drowsiness, shivering, skin itch, hypotension, and sweating. Overall, low-dose naloxone combined with opioids seemed to increase analgesia and reduce the risk of some certain opioid-related side effects during the perioperative period. The administration of low-dose naloxone might result in minor reductions in pain intensity.

The precise mechanisms of how low-dose naloxone contributed to decreased risk of some opioids' adverse events without affects opioids analgesic effect remain unknown. A low dose of naloxone may reduce opioid-related adverse effects and increase the analgesia effect by preventing a G protein coupling switch (Gi/o to Gs) of the mu opioid receptor [28]. Other mechanisms may include inhibiting Ca(2+) channel, improving inflammatory reaction, inhibiting the p38 mitogen-activated protein kinase, and mediating central kappa opioid [29–31].

Our review has several strengths. This meta-analysis and systematic review was prospectively registered and reported in consistent with PRISMA. We included an extensive scope of low-dose naloxone evaluated in randomized controlled studies as they provided the best evidence on the effectiveness and safety in clinical practice. We included 18 trials of low-dose naloxone during the perioperative period published up until 31 Day 2023. We used the Cochrane risk of bias tool to evaluate the level risk of bias and assessed the certainty of the evidence using the GRADE system.

Our review has limitations. First, although we included randomized controlled studies published in English, Chinese, and Korean, we could have missed some relevant trials. Second, the dose of naloxone was different between the included trials, which might lead to certain errors in the analysis results. Third, the study populations and types of surgeries varied among the included trials, which might limit the generalizability of the findings to specific patient populations or types of surgery. Fourth, the duration of follow-up for assessing adverse events was not specified, which could be important if some adverse events occur later in the postoperative period. In addition, not all results were collected in all studies.

Our results are some consistent with prior meta-analysis. In a prior meta-analysis [8] based on nine randomized controlled trials of 946 individuals, low-dose naloxone plays an important role in preventing postoperative nausea (RR: 0.80 and 95% CI: 0.67–0.95), while exhibiting no significant effects on postoperative vomiting (RR: 0.83 and 95% CI: 0.63–1.09). Compared with opioids used alone, low-dose naloxone did not significantly reduce visual analog scale pain scores (mean difference: −0.11 and 95% CI: 0.26–−0.05) in six studies. In another meta-analysis published in 2016 [9], 13 studies based on 1138 patients were included for review. In that review, the usage of naloxone in two trials was taken orally. Naloxone could significantly reduce the occurrence of pruritus (RR: 0.25 and 95% CI: 0.14–0.46), nausea (RR: 0.32 and 95% CI: 0.24–0.43), and vomiting (RR: 0.34 and 95% CI: 0.19–0.59). Compared with prior meta-analysis, our review included more trials, including large and recently published trials and substantially more patients. Our review included 1784 patients based on 18 trials. Besides, our review reported more opioid-related adverse events and used the GRADE system to assess the certainty of evidence.

Our review provided important information for weighing the potential benefits of low-dose naloxone combined with opioids during the perioperative period. Key messages included the likelihood that low-dose naloxone may decrease the risk of adverse events without weakening opioids' analgesia. This meta-analysis provided key evidence and recommendations for clinical practice. However, new trials should determine the optimal naloxone dose. Future trials should also endeavor to reduce bias.

In conclusion, this meta-analysis demonstrated that intravenous opioids with low-dose naloxone during the perioperative period were associated with a decreased risk of some opioid-related adverse events when compared with opioids alone. It might be clinically irrelevant reduction in pain score which needs further study. Our findings support the use of opioids with low-dose naloxone in the perioperation area.

## Figures and Tables

**Table 1:** Baseline characteristics of trials and participants.

Trials	Total sample	Operation name	Naloxone			Opioids			Outcomes
			No	Mean (sD) age (years):	Dose	Name	Mean (sD) age (years):	No	
Gan 1997	60	Hysterectomy	40	47 ± 12 50 ± 13	IV infusion: 0.25 or 1 mcg/kg/h	Morphine	51 ± 14	20	Nausea, vomiting, pruritus, respiratory depression
Koo 2001	72	Ocular plastic surgery	36	35.4 ± 14.2	PCA admixture: rate n/a	Fentanyl	34.5 ± 16.8	36	VRS, nausea, vomiting, pruritus, urinary retention
Cepeda 2002	166	Mixed	86	43.5 ± 10.9	PCA admixture: 0.5 mcg/kg/hr	Morphine	43.4 ± 9.6	80	Nausea, vomiting, sedation, urinary retention, pruritus
Sartain 2003	92	Hysterectomy	46	65 (60–79)	PCA admixture: 0.38 mcg/kg/hr	Morphine	72 (62–78)	46	VAS, nausea, vomiting, pruritus, sedation
Cepeda 2004	265	Mixed	136	42.9 ± 11.1	PCA admixture: 0.006–0.05 mcg/kg/hr	Morphine	41.4 ± 11.0	129	Nausea, vomiting, pruritus, sedation, urinary retention
Zhao 2005	55	Abdominal surgery	27	—	PCA admixture: 018 mcg/kg/hr	Morphine	—	28	VAS, nausea, vomiting, pruritus, dizziness
Yeh 2008	112	Hysterectomy	75	44.5 (23–66) 45.7 (23–65)	PCA admixture: 0.001–0.061 mcg/kg/hr	Morphine	44.8 (21–62)	37	Nausea, vomiting, pruritus, respiratory depression
Jia 2010	78	Cervical vertebrae surgery	60	54.2 ± 2.9 51.7 ± 3.3 53.2 ± 3.1	IV infusion: 0.05, 0.1, or 0.2 mcg/kg/hr	Tramadol	53.6 ± 2.4	18	VAS, vomiting
Xiao 2015	48	Open colorectal surgery	24	50 (23–64)	IV infusion: 0.25 mcg/kg/hr	Remifentanil	49 (25–65)	24	PONV
Zheng 2016	60	Elective laparoscopic cholecystectomy	30	18–55	PCA 0.42 mcg/kg/hr	Fentanyl	18–55	30	VAS, PONV
Koo 2017	61	Elective thyroid surgery	30	50 (24–71)	IV infusion: 0.05 mcg/kg/hr	Remifentanil	44 (24–75)	31	Nausea, dizziness, headache, drowsiness, shivering
Firouzian 2018	80	Open discectomy	40	36.63 ± 7.85	IV infusion: 0.25 mcg/kg/hr	Morphine	38.5 ± 8.96	40	VAS
Zou 2018	120	Caesarean section	90	21∼35	IV infusion: 0.10–0.30 mcg/kg/hr	Butorphanol	21∼35	30	VAS, somnolence, dizzy, nausea, vomit, respiratory depression, skin itch
Lin 2019	69	Video-assisted thoracoscopic resection of lung cancer	35	55.46 ± 8.65	PCA 0.05 mcg/kg/h	Sufentanil	55.46 ± 8.65	34	Nausea, vomiting
Makarem 2020	50	Elective hysterectomy	25	50.56 ± 7.0	IV infusion: 0.25 mcg/kg/hr	Remifentanil	50.32 ± 6.6	25	VAS, PONV, shivering
Omar 2021	90	Elective unilateral-modified radical mastectomy	45	48.9 ± 1.99	IV infusion: 100 ng	Fentanyl	49.3 ± 1.37	45	Hypotension, PONV
Qian 2022	186	Elective gynecological laparoscopic surgery	93	48.8 ± 10.4	IV infusion: 0.5 mcg/kg/hr	Sufentanil	50.2 ± 9.8	93	VAS, PONV, cough, respiratory depression
Yang 2022	120	Elective gynecological laparoscopic surgery	90	37.37 ± 1.84 39.30 ± 1.79 36.70 ± 1.68	IV infusion: 0.2 or 0.4 or 0.6 mcg/kg/h	Sufentanil, butorphanol	37.30 ± 2.06	30	Skin itch, dizziness, sweating

Abbreviations: PCA, patient-controlled analgesia; PONV, postoperative nausea and vomiting; VAS, visual analog scale.

**Table 2:** Summary of findings and quality of evidence for safety.

Adverse event	Summary of findings		Certainty of evidence					Overall certainty of evidence
	No of participants (no. of trials)	Relative risk⁣^∗^ (95% CI)	Study limitations	Inconsistency	Indirectness	Imprecision	Reporting bias	
Nausea	1072 (10)	0.82 (0.70–0.96)	Not downgraded	Not downgraded	Not downgraded	Not downgraded	Not downgraded	High
Vomiting	1089 (10)	0.86 (0.70–1.06)	Not downgraded	Not downgraded	Not downgraded	Downgraded^§^	Not downgraded	Moderate
PONV	434 (5)	0.58 (0.40–0.80)	Not downgraded	Not downgraded	Not downgraded	Not downgraded	Not downgraded	High
Pruritus	822 (7)	0.75 (0.55–1.02)	Not downgraded	Not downgraded	Not downgraded	Downgraded^§^	Not downgraded	Moderate
Urinary retention	503 (3)	0.56 (0.27–1.17)	Not downgraded	Not downgraded	Not downgraded	Downgraded^§^	Not downgraded	Moderate
Sedation	523 (3)	0.98 (0.85–1.13)	Not downgraded	Not downgraded	Not downgraded	Not downgraded	Not downgraded	High
Dizziness	356 (4)	0.86 (0.33–2.23)	Downgraded^‡^	Not downgraded	Not downgraded	Downgraded^§^	Not downgraded	Low
Respiratory depression	478 (4)	0.48 (0.09–2.59)	Downgraded^‡^	Not downgraded	Not downgraded	Downgraded^§^	Not downgraded	Low
Headache	61 (1)	0.88 (0.30–2.63)	Not downgraded	Not downgraded^¶^	Not downgraded	Downgraded^§^	Not downgraded^¶^	Moderate
Drowsiness	61 (1)	1.03 (0.15–6.89)	Not downgraded	Not downgraded^¶^	Not downgraded	Downgraded^§^	Not downgraded^¶^	Moderate
Shivering	111 (2)	0.39 (0.13–1.18)	Not downgraded	Not downgraded	Not downgraded	Downgraded^§^	Not downgraded	Moderate
Somnolence	120 (1)	0.29 (0.14–0.58)	Downgraded^†^	Not downgraded	Not downgraded	Not downgraded	Not downgraded	Low
Skin itch	240 (2)	0.83 (0.16–4.20)	Downgraded^†^	Not downgraded	Not downgraded	Downgraded^§^	Not downgraded	Very low
Hypotension	90 (1)	1.00 (0.15–6.81)	Not downgraded	Not downgraded^¶^	Not downgraded	Downgraded^§^	Not downgraded^¶^	Moderate
Cough	186 (1)	0.52 (0.30–0.90)	Not downgraded	Not downgraded^¶^	Not downgraded	Not downgraded	Not downgraded^¶^	High
Sweating	120 (1)	0.67 (0.06–7.18)	Not downgraded	Not downgraded^¶^	Not downgraded	Downgraded^§^	Not downgraded^¶^	Moderate

⁣^∗^Data are relative risk for adverse events. A relative risk < 1 indicates that effects favor naxolone combined with opioids compared with control.

^†^Downgraded two levels: > 50% of the participants were from studies at high risk of bias.

^‡^Downgraded one level: > 25% but < 50% of the participants were from studies at high risk of bias.

^¶^Not downgraded: could not be determined with one study.

^§^Downgraded one level: limits of the 95% confidence interval crossed the null.

## Data Availability

The data used to support the findings of this study are included within the article.
